# Extirpation of the Stomach of a Cat

**Published:** 1895-04

**Authors:** 


### Extirpation of the Stomach of a Cat.

At a recent meeting of the Paris Biological Society reported in the *Gazette Medicale de Paris*, M. M. Parchon and Corvallo exhibited a cat from which they had removed the stomach, and they detailed the results of the operation. The general nutrition was normal, the three species of foods—albuminoids, carbo-hydrate and fats were well digested; the digestion of milk, however, was faulty. The animal thrived best on a pap of milk, rice, flour, and yolk of an egg. Cooked meat was perfectly digested, raw meat imperfectly. Professor Dasht observed that we know the gastric juice produces changes in meat similar to those from cooking.

